# Interaction and Antioxidant Functions of Lactoferrin–*Polygonatum sibiricum* Polysaccharide Complexes at Different pH

**DOI:** 10.1155/ijfo/8381915

**Published:** 2026-04-17

**Authors:** Runru Xiang, Duoduo Zhang, Shunjie Kang, Jiwei Ding, Qin Shu, Guo Hao, Tao Ma, Yongfeng Liu

**Affiliations:** ^1^ College of Food Engineering and Nutritional Science, Shaanxi Normal University, Xi’an, 710119, Shaanxi, China, snnu.edu.cn; ^2^ Shaanxi Goat Milk Product Quality Supervision and Inspection Center, Fuping, 711700, Shaanxi, China

**Keywords:** antioxidant activity, interaction, lactoferrin, *Polygonatum sibiricum* polysaccharide

## Abstract

The interactions between proteins and polysaccharides are of great significance in the development of innovative functional foods. This study aimed to systematically explore the interaction mechanisms between lactoferrin (LF) and *Polygonatum sibiricum* polysaccharide (PSP) and evaluate their antioxidant activities. The interactions between LF and PSP within the pH range of 3.0–8.0 were characterized by multispectroscopic techniques such as fluorescence spectroscopy and infrared spectroscopy. It was found that the main driving force of their interaction was electrostatic adsorption. The binding of PSP induced changes in the secondary and tertiary structures of LF, thereby enhancing the antioxidant activity of LF. After adding PSP, the DPPH scavenging rate reached up to 81.79%, and the ABTS scavenging rate reached 93.18%. These findings provide new insights at the molecular level into protein–polysaccharide interactions and demonstrate the potential of LF‐PSP complexes as natural antioxidants, which may facilitate the design of innovative functional dairy products.

## 1. Introduction

In food systems, proteins and polysaccharides are two common and important biomacromolecules. Their interactions are critical determinants of the system’s thickening properties, shape formation, processing stability, and texture maintenance [1]. The functional properties displayed by protein–polysaccharide complexes are collectively influenced by the molecular characteristics and interaction patterns of these two biomacromolecules [2]. However, multiple factors, including pH, ionic strength, and temperature, impact their interactions [3]. Therefore, understanding and controlling the effects of these factors on interactions is essential for designing novel food systems.

Lactoferrin (LF) is a glycoprotein with strong iron‐binding capacity and a molecular weight of approximately 80 kDa. It possesses outstanding nutritional and biological activity, making it highly promising for applications in the food and healthcare sectors [4]. Research indicates that LF possesses multiple physiological functions, including preventing pathogen infection and promoting immunity, while also exhibiting significant antioxidant potential [5, 6]. However, LF’s physicochemical properties are highly unstable and susceptible to environmental pH and processing conditions, resulting in low bioavailability and poor absorption efficiency. This hinders the full realization of its antioxidant capabilities [7]. Therefore, to address the need for iron fortification in certain foods and to improve the bioavailability of LF, it is essential to study the synergistic effect of LF with other natural antioxidants. In multicomponent systems, antioxidants may enhance each other through synergistic interactions, resulting in improved antioxidant activity.

*Polygonatum sibiricum,* a species of the genus Pelargonium, is valued for its dual applications in medicine and food [8]. Polysaccharides derived from *P. sibiricum* (PSP) represent the essential functional elements of *P. sibiricum* [9]. The PSP demonstrate various biological activities, such as acting as antioxidants, providing antiaging effects, and serving as natural antioxidants [10]. The unique properties of PSP, particularly their antioxidant and anti‐inflammatory characteristics, suggest that they could play a significant role in the treatment of diseases associated with oxidative stress [11–13]. The antioxidant capabilities of PSP have been confirmed via chemical analyses, along with in vitro and in vivo experiments, showing that PSP possess strong scavenging activity against 2,2′‐azino‐bis‐(3‐ethylbenzothiazoline‐6‐sulfonic acid) (ABTS), DPPH, and hydroxyl radicals [10]. Therefore, investigating the interaction between LF and the natural antioxidant *P. sibiricum* polysaccharides (PSPs) not only holds promise for enhancing the antioxidant activity and bioavailability of both compounds but may also provide new evidence for developing novel antioxidant functional foods.

This study investigated the interactions between LF and PSP under varying pH conditions through surface charge, spectroscopic analysis, physical stability, and microstructural characterization. It further characterized the surface hydrophobicity and antioxidant activity of the LF‐PSP complex. These findings establish a foundation for improving the bioavailability of LF‐PSP and provide valuable perspectives for creating functional dairy products.

## 2. Materials and Methods

### 2.1. Materials

LF (95%) and PSP (70%) were both purchased from Shanghai Yuanye Biotechnology Co., Ltd.

### 2.2. Preparation of the Complexes

Disperse LF and PSP powders separately in water and then stir for 2 hours to prepare 1% LF solution and 0.1% PSP solution. Subsequently, adjust the pH of the LF and PSP solutions to 3.0, 4.0, 5.0, 6.0, 7.0, and 8.0, respectively. At each identical pH level, the two solutions were combined in equal volumes and stirred for 2 h to create LF‐PSP complexes. The LF solution at the same pH served as the control.

### 2.3. Particle Size and Zeta Potential

Particle size and zeta potential values of LF and LF‐PSP solutions at pH 3.0–8.0 were determined and analyzed using a laser particle size zeta potential meter (NanoBrook 90 Plus PALS), respectively.

### 2.4. Fluorescence Analysis

Fluorescence spectra intrinsic to LF and the LF‐PSP complex were obtained using a fluorescence spectrophotometer (RF‐6000, Shimadzu, Japan) at an excitation wavelength of 280 nm, covering an emission wavelength range from 300 to 500 nm.

### 2.5. UV–Visible Spectrum Analysis

The UV–visible spectrophotometer (T9 CS, Beijing Persee, China) was used to determine the UV absorption spectra of the LF and LF‐PSP complex samples in the range of 260–400 nm and was analyzed.

### 2.6. Fourier Transform Infrared (FTIR) Analysis

The infrared spectra of LF and LF‐PSP complexes were measured in the frequency range of 4000–400 cm^−1^using a FTIR spectrometer (Invenio S, Bruker, UK) for determination. The secondary structures of LF and LF‐PSP complexes were further analyzed using PeakFit 4.12 software.

### 2.7. SDS–PAGE Analysis

LF and LF‐PSP complexes were analyzed using SDS–PAGE [14]. Following thorough mixing with the loading buffer and denaturation at 95°C for 4 min, the samples were subjected to SDS–PAGE. Then, the samples were stained and observed using a rapid staining solution of Coomassie Brilliant Blue.

### 2.8. Scanning Electron Microscope (SEM) Analysis

Using a SEM (TM3000, HITACHI, Tokyo, Japan) at 15 kV high voltage, the sample microstructure was observed at 600x magnification.

### 2.9. Surface Hydrophobicity Determination

The surface hydrophobicity (H_0_) was evaluated using an 8‐anilino‐1‐naphthalenesulfonic acid (ANS) fluorescent probe and a fluorescence spectrophotometer. The samples (4 mL) were mixed with 20 μL of ANS (8.0 mmol/L), and the fluorescence measurements of LF and LF‐PSP complexes were performed under the excitation and emission slits of 5 nm, emission wavelengths of 400–600 nm, and excitation wavelengths of 365 nm [15].

### 2.10. Physical Stability

LF and LF‐PSP solutions of different pH were placed in an emulsion stability analyzer (Turbiscan tower) for 10,000 s and scanned every 120 s to obtain the kinetic instability index TSI to describe the physical stability of the complexes.

### 2.11. Free Sulfhydryl Groups

The determination of free sulfhydryl groups was based on the method of Yang et al. [16], with slight modifications. The calculation formula is as follows:

(1) free SH contentsμmol/g=73.53×A412×DC.

The absorbance measured at 412 nm is represented by *A*_412_, while *C* signifies the concentration of protein (mg/mL) and *D* symbolizes the dilution factor.

### 2.12. Antioxidant Properties

#### 2.12.1. DPPH Scavenging Rate

The DPPH scavenging activity of each sample was assessed based on the methodology of Jakubczyk et al. [17]. A volume of 1.5 mL from the sample solution was carefully measured and combined with 3 mL of a 200 μM DPPH solution. The absorbance of the resulting mixture was measured at 517 nm after allowing it to react, shielding it from light for 30 min. The calculation formula is as follows:

(2) DPPH scavenging rate%=Ac−AsAc×%,

where *A*_*s*_ and *A*_*c*_ represent the absorbance values of the DPPH solution with and without the sample at 517 nm, respectively.

#### 2.12.2. ABTS Scavenging Rate

ABTS solution stock solution was prepared by weighing 200.0 mg of ABTS and 34.4 mg of potassium persulfate, dissolving them in 50.0 mL of distilled water, shaking well, and then storing them for 24 h at room temperature away from light. The ABTS solution must be diluted to the appropriate concentration before use. Combine 0.15 mL of supernatant with 0.85 mL of the diluted ABTS solution, allow the mixture to react for 10 min in a dark environment, and then measure the absorbance at a wavelength of 734 nm [9]. Clearance was calculated by the following equation:

(3) ABTS scavenging rate%=Ac−AsAc×%.

The absorbance values for the sample group and the control group are denoted by *A*_*s*_ and *A*_*c*_, respectively.

#### 2.12.3. Hydroxyl Radical Scavenging Rate

A 6 mmol/L FeSO_4_ solution, 6 mmol/L salicylic acid solution, and 6 mmol/L H_2_O_2_ solution were prepared. Accurately pipetted 2 mL of the sample into the tube, then added 2 mL of FeSO4 solution, 2 mL of salicylic acid solution, 2 mL of H_2_O_2_ solution, shook well, and carried out at 25°C for 30 min in the dark, measured the absorbance value at 510 nm (*A*_1_), and used deionized water instead of H_2_O_2_ solution to measure the absorbance value at 510 nm (*A*_0_), and the absorbance value at 510 nm was measured with deionized water instead of sample (*A*_2_). The calculation formula is as follows:

(4) hydroxyl scavenging rate %=A2−A1+A0A2×%.

#### 2.12.4. Reducing Power Determination

Iron reduction antioxidant capacity determination was carried out by referring to the method described by Fan et al. [19] with slight modifications. After adding 2 mL of polysaccharide samples with different concentrations in a test tube, 2 mL of 1% potassium ferricyanide solution and PBS buffer pH 6.6 were added, respectively, and heated in a water bath for 20 min at 50°C. Following this, 2 mL of 10% trichloroacetic acid solution was incorporated and mixed well, and then, the water bath was set at 50°C for 10 min, and then after the temperature was lowered to room temperature, the absorbance of the mixture was assessed at a wavelength of 700 nm. The reducing capacity was calculated according to the following formula:

(5) reducing power=A2−A1,

where *A*_1_ and *A*_2_ represent the absorbance values of the background and sample groups, respectively.

### 2.13. Statistical Analysis

All experimental data were measured three times in parallel, and the mean ± standard deviation was calculated if not otherwise indicated. The data were statistically analyzed using SPSS Statistics 26 software. Different letters indicate statistically significant differences between variables (*p* < 0.05).

## 3. Results and Discussion

### 3.1. Zeta Potential and Particle Size

The zeta potential serves as an important measure for assessing the interactions among biopolymers [20]. Consequently, we evaluated the zeta potential values of LF, PSP, and the LF‐PSP complex across various pH levels ranging from 3.0 to 8.0. The presence of hydroxyl groups within the PSP molecule results in it carrying a negative charge across all chosen pH levels (Figure 1(a)) [13]. As the pH value decreases, there is a continuous decline in the absolute value of the zeta potential, which changes from an initial measurement of 30.36 mV down to 0.35 mV. Conversely, due to its high basicity, LF exhibits a positive zeta potential when the pH is below its isoelectric point (pI) of approximately 9.00 [22]. In contrast, when the pH exceeds its isoelectric point (pI), LF carries a negative charge. The selected pH ranges are all below the LF isoelectric point, so as shown in Figure 1(b), LF is positively charged at pH 3.00–8.00. Therefore, by adjusting the pH of the samples, the electrostatic interactions between them can be controlled. The zeta potential of the LF‐PSP complex (Figure 1(c)) exhibits significant pH dependence, gradually decreasing with increasing pH. This potential shift may result from electrostatic interactions between negatively charged groups on PSP and positively charged amino groups on the LF surface [23]. Specifically, the complex exhibits a positive charge at pH ≤ 6 and a negative charge at pH ≥ 7. This shift in potential from positive to negative may be attributed to the isoelectric point of LF shifting to a lower pH value following polysaccharide modification, further indicating the occurrence of electrostatic interactions between LF and PSP [24].

FIGURE 1Zeta potential (mV) of PSP (a), LF (b), and LF‐PSP complexes (c); mean grain size of LF and LF‐PSP complexes (d); size distribution of LF (e) and LF‐PSP complexes (f); the red dashed box in (c) indicates the shift in net surface charge occurring at a pH of approximately 6.0. This shift results from the electrostatic neutralization between LF and PSP, causing a displacement in the net surface charge.

**Figure:**
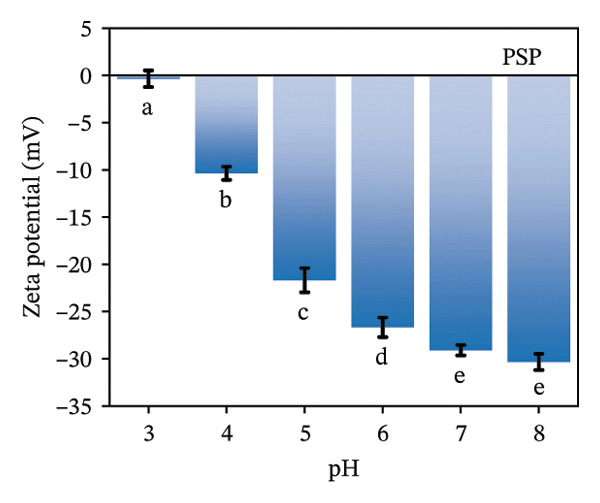
(a)

**Figure:**
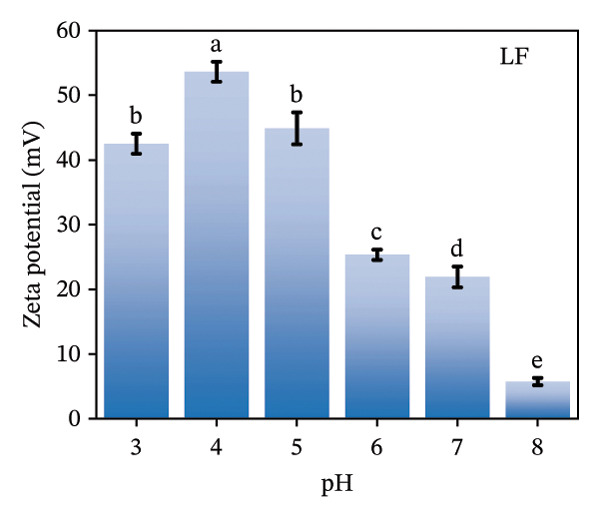
(b)

**Figure:**
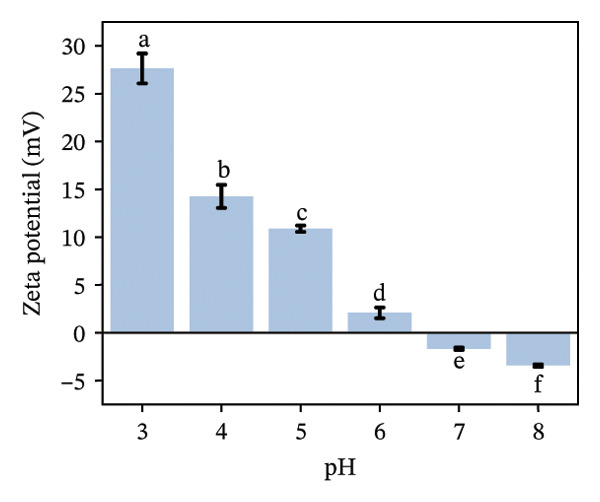
(c)

**Figure:**
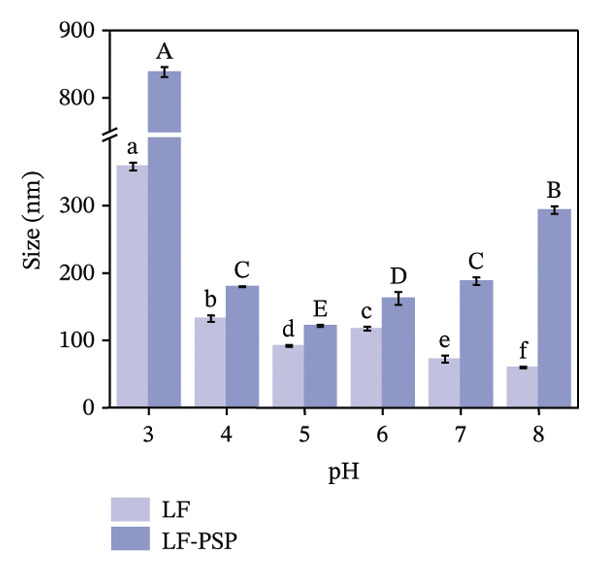
(d)

**Figure:**
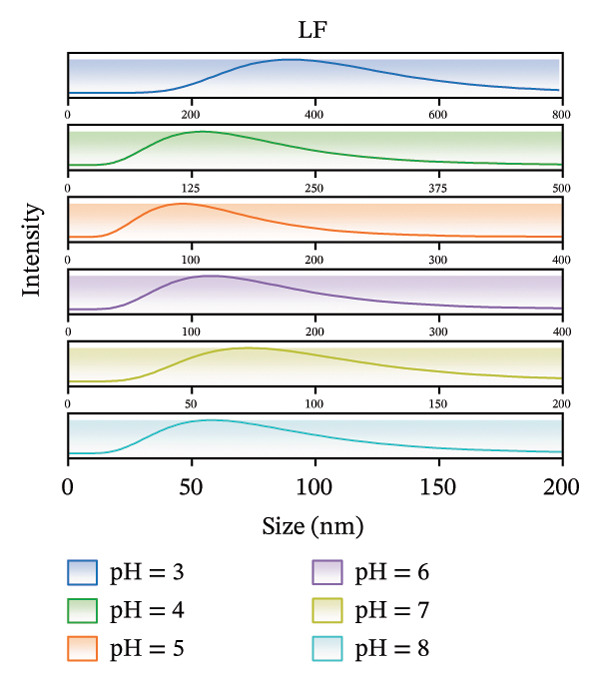
(e)

**Figure:**
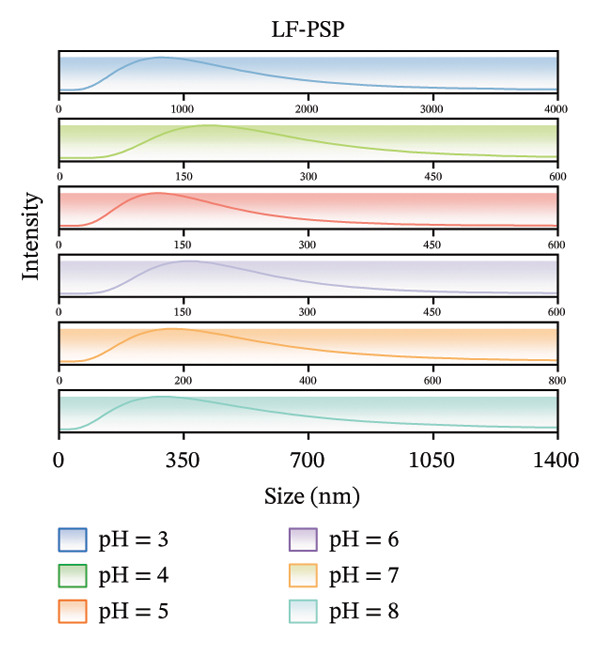
(f)

Figures 1(d), 1(e), 1(f) shows the average sizes of particles in LF and LF‐PSP complexes and their particle size distributions, respectively. As the pH increased from 3.0 to 8.0, the sizes of the LF and LF‐PSP complexes exhibited an irregular decreasing pattern. The largest particle sizes were observed at pH 3.0, with LF measuring 357.90 nm and LF‐PSP measuring 838.18 nm. LF exhibited a minimum particle size of 59.47 nm at pH 8.0, while LF‐PSP reached its minimum particle size of 121.36 nm at pH 5.0. In addition to this, the results showed that the sizes of the LF particles were less than those of the LF‐PSP complexes, which may be due to the incorporation of PSP interacting with LF to form larger complex particles or due to the fact that their interactions lead to LF aggregation.

### 3.2. Fluorescence Analysis

Intrinsic fluorescence spectroscopy is often utilized to identify alterations in polarity within the microenvironment of hydrophobic amino acid residues, making it a valuable technique for studying the tertiary structure of proteins [25]. The maximum absorption peak of LF occurs at 327 nm in the pH range of 4.0–8.0, which shifted to 339 nm when the pH was decreased to 3.0 (Figure 2(a)). It is in agreement with the results of Yang et al., which suggests that more tryptophan is exposed to the solvent as the pH decreases [26]. Furthermore, the fluorescence intensity of LF exhibited inconsistent variations at varying pH levels, suggesting a possible change in the tertiary structure of the protein due to alterations in pH. As illustrated in Figure 2(b), the LF‐PSP complex shows a noticeably lower fluorescence intensity and a shift toward red in its peak emission wavelength when contrasted with LF. Research conducted earlier has suggested that an increase in fluorescence intensity generally signifies the incorporation of hydrophobic amino acid residues, particularly tryptophan, within the core hydrophobic areas of protein structures. However, a decrease in fluorescence intensity frequently indicates either a partial or total unfolding of the protein’s tertiary structure, which results in tryptophan residues becoming exposed to a watery environment [27, 28]. Consequently, one may deduce that the fluorescence quenching noted in this experiment could be linked to the protein structure’s unfolding prompted by the interaction occurring between LF and PSP. This alteration in structure might additionally promote the reformation of hydrophobic areas via hydrophobic interactions, leading to a reduction in fluorescence intensity. In summary, the inclusion of PSP not only enhanced the fluorescence quenching of LF but also significantly altered the tertiary structure of LF.

FIGURE 2Fluorescence spectrum analysis of LF (a) and LF‐PSP (b) complexes and UV–visible absorption spectrum analysis of LF (c) and LF‐PSP (d) complexes.

**Figure:**
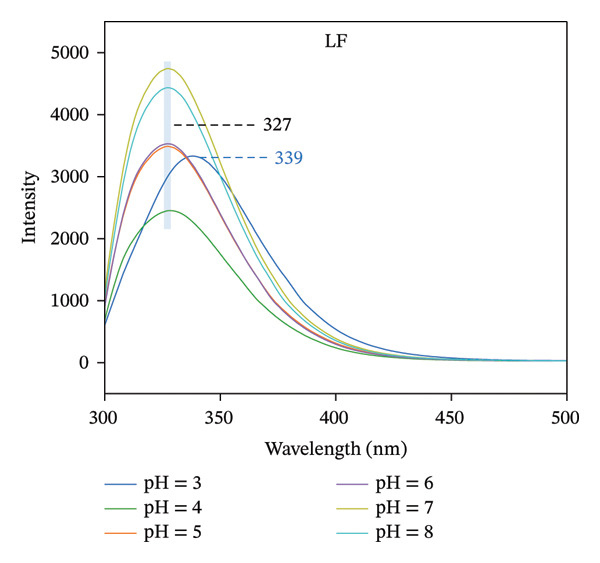
(a)

**Figure:**
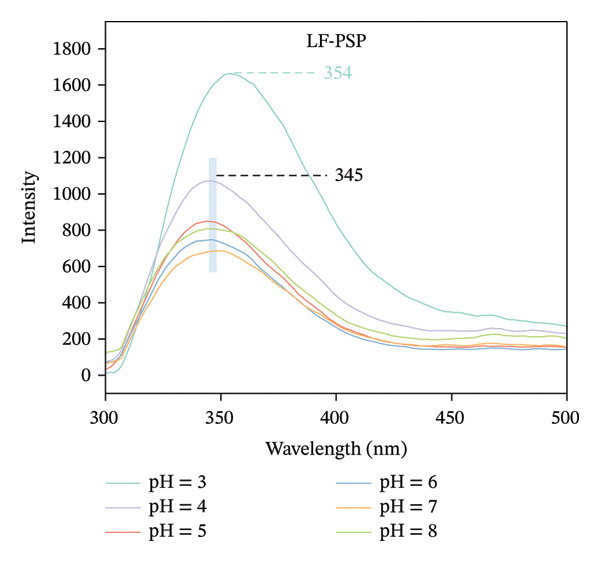
(b)

**Figure:**
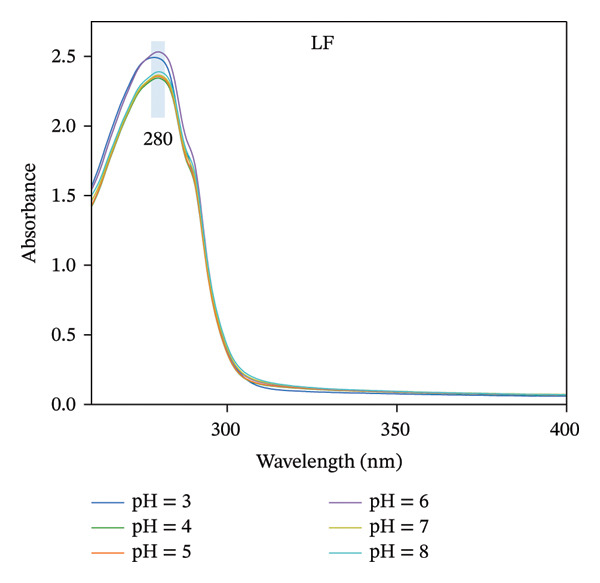
(c)

**Figure:**
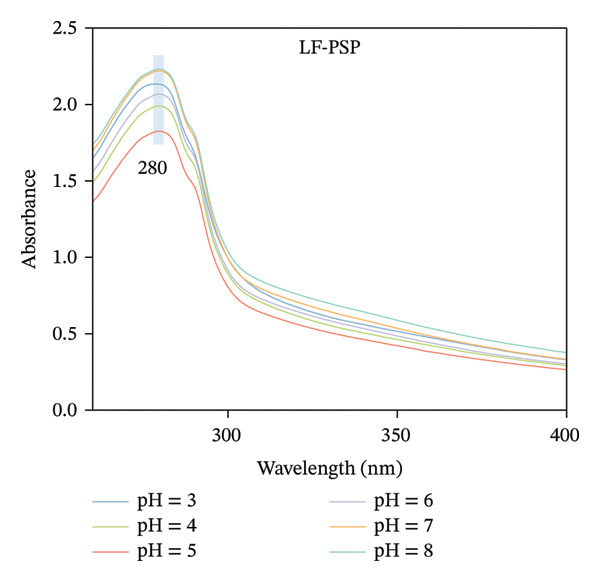
(d)

### 3.3. UV–Vis Absorption Analysis

Because amino acids in protein side chains show significant absorption peaks in the near‐ultraviolet range, ultraviolet absorption spectroscopy can be utilized to investigate how LF‐PSP interactions impact the tertiary structure of proteins [29]. As illustrated in Figures 2(c), 2(d), LF and LF‐PSP exhibit their strongest characteristic peaks near 280 nm, and the absorption peak intensity of LF shows no significant change with pH variation, and the UV absorption intensity of LF was reduced after binding with PSP and correlated with pH. This may be related to the electrostatic binding between LF and PSP. These interactions induce structural rearrangement in the protein, burying hydrophobic amino acid residues originally exposed to the polar environment within its internal nonpolar regions, ultimately increasing its surface hydrophobicity [30].

### 3.4. FTIR Analysis

FTIR is a commonly used technique for evaluating changes in protein secondary structure. The amide I band (1700–1600 cm^−1^, primarily originating from C=O stretching vibrations) and the amide II band (1575–1480 cm^−1^, primarily originating from N‐H bending vibrations) are typically used together for precise secondary structure identification. The amide III band (1300–1225 cm^−1^, originating from C‐N stretching and N‐H bending) also frequently serves as an auxiliary analytical region. Additionally, stretching vibration peaks of free hydroxyl groups (‐OH) typically appear around 3300–3170 cm^−1^ [31]. All samples had a broad peak at 3000–3700 cm^−1^, which was caused by the stretching vibration of the free hydroxyl group or the deformation vibration of N‐H, indicating the presence of hydrogen bonding in the system [32]. The wavelength of LF amide I exhibited a certain shift at different pH (Figure 3(a)). As the pH increased from 3.0 to 8.0, the wavelength of LF amide I gradually shifted from 1640.40 to 1656.62 cm^−1^, which may be attributed to the stretching vibration of C=O, which implies that LF’s secondary structure has changed. In contrast, the wavelength of LF amide II did not change much, 1538.41 cm^−1^at pH 3.00–4.00 and 1054.73 cm^−1^at pH 5.0–8.0. Compared with LF, the addition of PSP induced wavelength shifts in both the amide I and II bands of LF (Figure 3(b)). Specifically, the LF‐PSP complex exhibited a red shift in the amide I band at pH 3.0–6.0 and a blue shift at pH 7.0–8.0, while the amide II band underwent a red shift across the tested pH range, indicating a modification of the LF secondary structure by PSP [33].

FIGURE 3FTIR of LF (a) and LLF‐PSP (b) complexes; secondary structure of LF (c) and LLF‐PSP (d) complexes.

**Figure:**
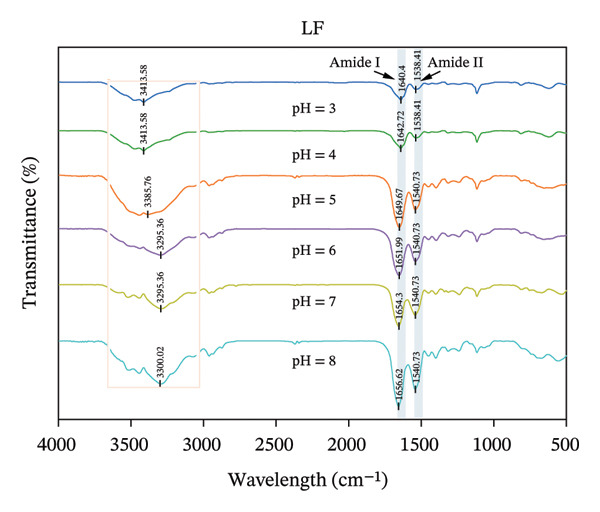
(a)

**Figure:**
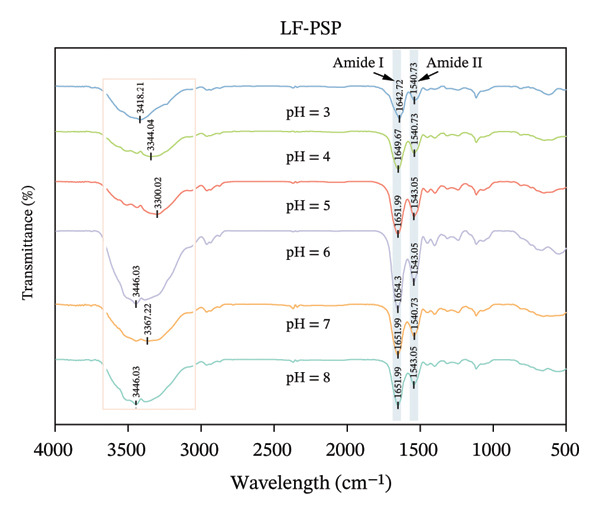
(b)

**Figure:**
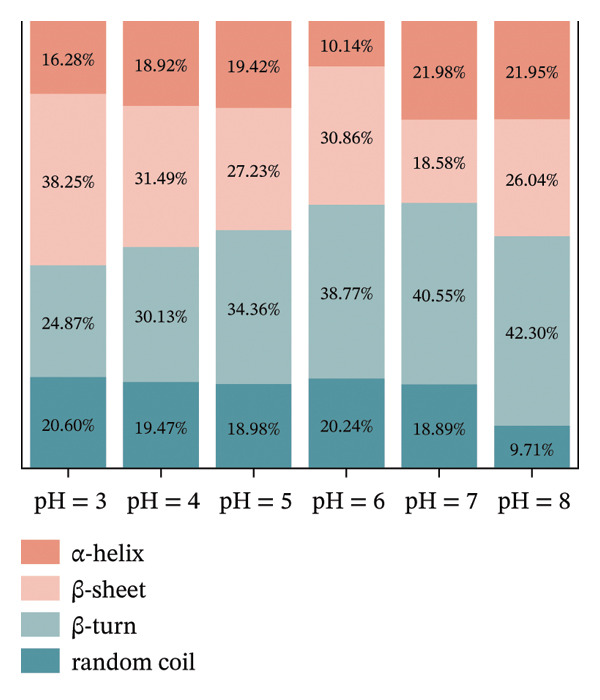
(c)

**Figure:**
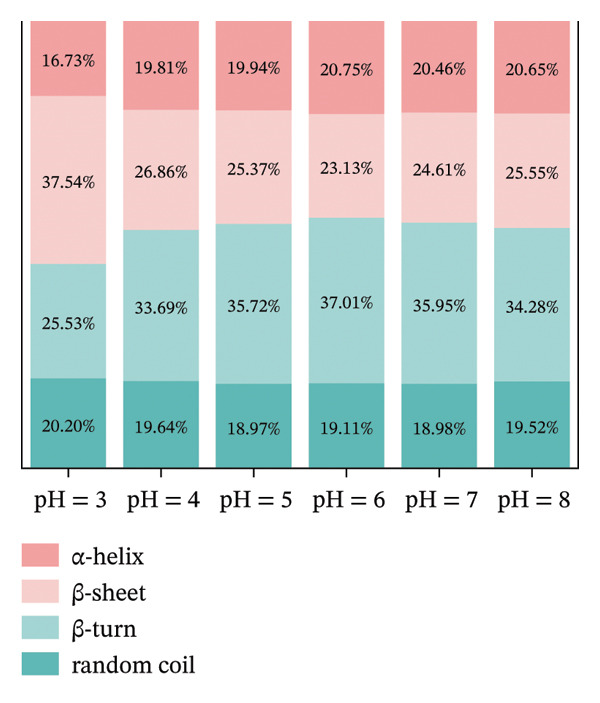
(d)

In order to gain a deeper understanding of the alterations in the secondary structure content of LF and its complex with polysaccharide (LF‐PSP), this study utilized PeakFit 4.12 software. This analytical tool was instrumental in quantifying the changes in secondary structure content for both LF and the LF‐PSP complex across various pH levels. As illustrated in Figure 3(c), LF’s secondary structure exhibited irregular changes at varying pH levels. Specifically, the proportion of β‐turns rose as pH increased, while there was an inconsistent decline in the amount of β‐sheets as pH levels rose. Typically, the α‐helix and β‐sheet formations form the stable core of the protein chain and play a crucial role in preserving conformational stability, while the irregularly curled structure grants the protein molecular flexibility [34]. Therefore, the rigidity of LF decreases upon pH action. The incorporation of PSP significantly altered the secondary structure of LF. In addition to this, the LF‐PSP complex exhibited lower β‐turn content than LF, while its α‐helix content exceeded that of LF across the pH range of 3.0–7.0. This indicates that PSP incorporation enhances the flexibility of LF. While the irregular coil provided flexibility, the content of the random coil of LF‐PSP also decreased with the addition of PSP, which indicated that LF‐PSP was more stable than LF [35].

### 3.5. The SDS–PAGE Analysis

**FIGURE 4:**
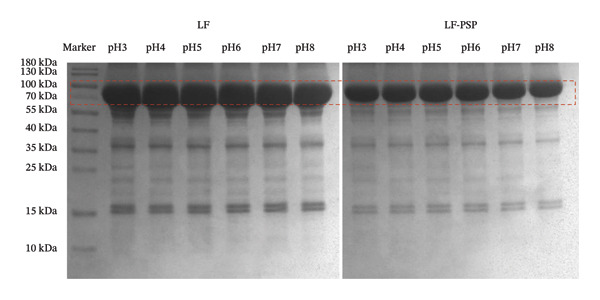
SDS–PAGE profiles of LF and LF‐PSP complexes with different pH; both LF and LF‐PSP complexes exhibit distinct bands near 80 kDa.

The results obtained from SDS–PAGE analysis for LF and LF‐PSP complexes at various pH levels are presented in Figure 4. In this context, it is important to note that both SDS and mercaptoethanol are recognized for their ability to disrupt noncovalent interactions as well as disulfide bonds. Consequently, the presence of covalent coupling within the complexes can be effectively assessed through the process of electrophoresis, allowing for a clear understanding of the interactions occurring within the samples under investigation [36]. The SDS–PAGE showed a strong band for LF near 80 kDa, consistent with its established molecular weight. Conversely, the LF‐PSP complex has a narrower band near 80 kDa and a lighter overall band color, further suggesting that the incorporation of PSP has altered the structure of LF.

### 3.6. SEM Analysis

**FIGURE 5:**
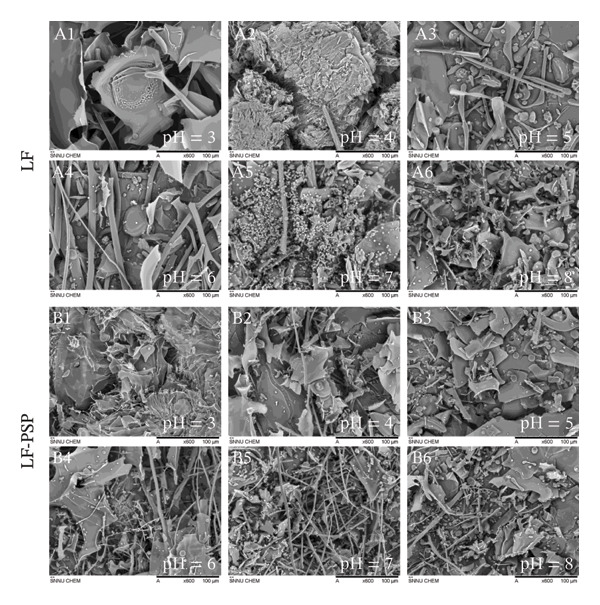
The SEM microstructure of LF (A1–A6) and LF‐PSP (B1–B6) complexes with different pH.

The microstructures of LF and LF‐PSP complex particles across various pH levels are illustrated in Figure 5. Following the freeze‐drying procedure, LF demonstrated clustering of particles, with spherical particles grouped closely and further twisted into three‐dimensional structures [11]. Natural LF exhibited disorganized lamellar, striped, and spherical structures [31]. The structural differences of LF at different pH levels were obvious (Figure 5 A1–A6). At pH 3.00–4.00, LF was mainly lamellar–lamellar, and as pH increased, LF was gradually dominated by a combination of strips and spheres, especially at pH 7.0, where spherical structures were particularly obvious, so it can be posited that the alteration in pH influenced the structure of LF to some degree. While PSP was mainly fragmented, accompanie d by a small number of bands, its clear surface structure showed a smooth and irregular morphology [33]. Therefore, the microstructure features of the LF‐PSP complex also predominantly exhibit layered, banded, and spherical structures (Figure 5 B1–B6). Specifically, at pH 3.0 to 5.0, the complex predominantly exhibits a lamellar morphology with numerous small spherical particles distributed on its surface. In contrast, at pH 6.0 to 8.0, the complex adopts a band‐like structure similar to LF, also bearing numerous small spherical particles on its surface.

### 3.7. Surface Hydrophobicity Analysis

Surface hydrophobicity serves as a key indicator for evaluating the distribution of hydrophobic groups and conformational changes in proteins [1]. ANS, as a fluorescent probe, exhibits fluorescence intensity correlated with the exposure of hydrophobic groups in folded intermediates to the solvent. Consequently, it is frequently employed to detect surface hydrophobicity in proteins [40]. The surface hydrophobicity of LF at various pH levels is illustrated in Figure 6(a). Notably, LF exhibited significantly greater surface hydrophobicity at pH 3.0 compared to other pH levels. This phenomenon may be attributed to the enhanced exposure of hydrophobic amino acid residues, such as leucine, isoleucine, and phenylalanine, at this particular pH, which subsequently increases the availability of binding sites for the ANS fluorescent probe. However, the surface hydrophobicity of the complex is significantly lower than that of LF. This could result from the interaction of LF with PSP, which conceals the hydrophobic residues on the surface of the protein, or it might be that the aggregation induced by PSP hinders the exposure of specific hydrophobic groups within the protein molecule [28]. A recent study also demonstrates that polysaccharides suppress the exposure of LF hydrophobic groups to hydrophilic environments, thereby reducing the surface hydrophobicity of the complex [31].

FIGURE 6Surface hydrophobicity of LF (a) and LF‐PSP (b) complexes with different pH; physical stability of LF‐PSP complexes (c); SH group content of LF and LF‐PSP complexes (d).

**Figure:**
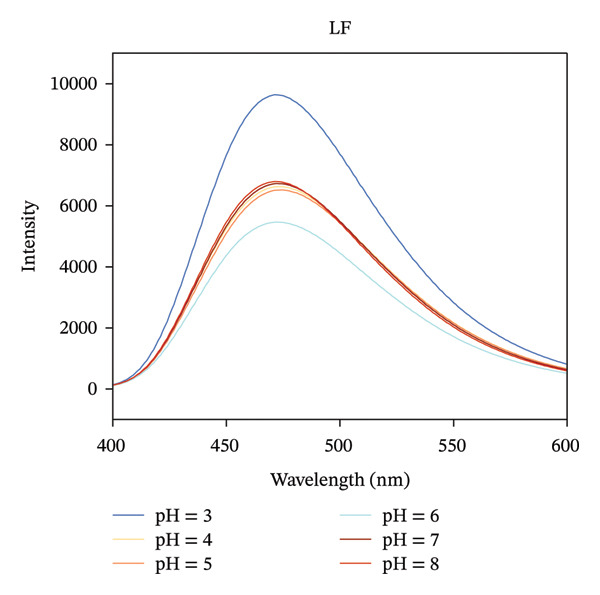
(a)

**Figure:**
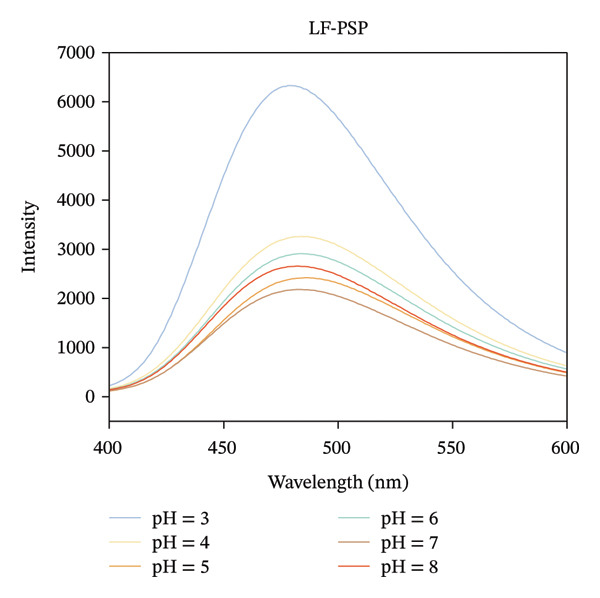
(b)

**Figure:**
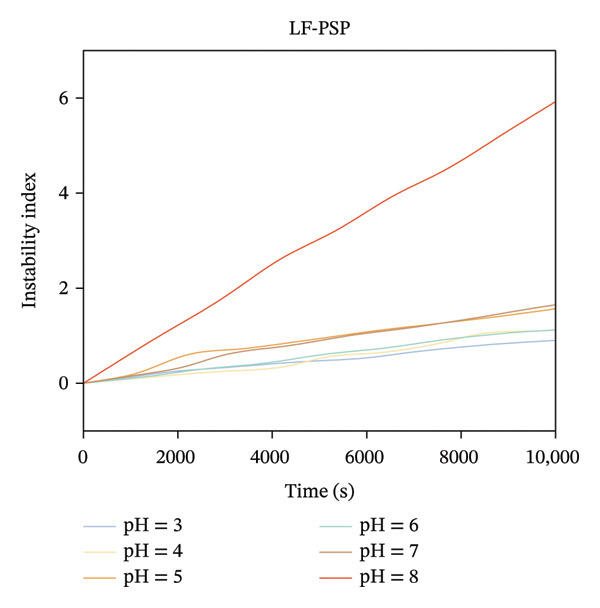
(c)

**Figure:**
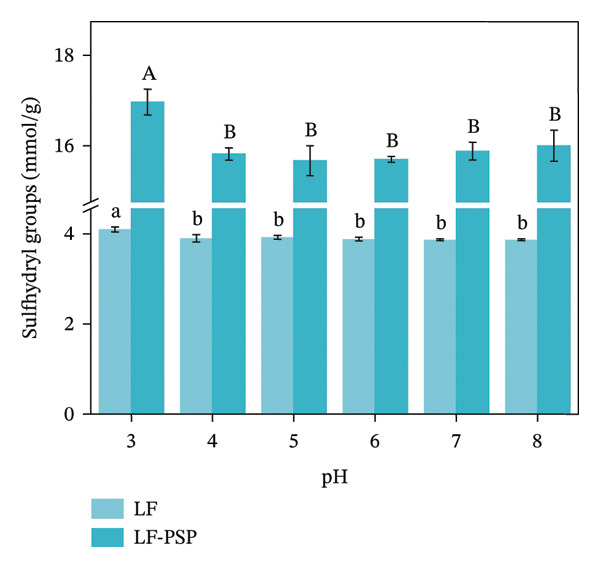
(d)

### 3.8. Physical Stability Analysis

This study employed an emulsion stabilizer to accelerate the flocculation and sedimentation processes within the LF‐PSP composite while monitoring changes in its transmittance. This enabled the calculation of the composite’s instability index under varying pH conditions [8]. As illustrated in Figure 6(b), the instability index for all composites exhibited a dynamic increase over time, indicating a continuous escalation of system instability. Concurrently, this index varied with pH conditions, confirming pH as a key factor influencing the composite’s physical stability. A clear negative correlation exists between physical stability and the instability index.

### 3.9. Free Sulfhydryl Content Analysis

Sulfhydryl groups play a crucial role as reactive participants in proteins. They are instrumental in preserving the secondary and tertiary structures of these proteins and are also significantly linked to their functional characteristics, such as antioxidant capacity and gel formation. Consequently, they influence the ultimate functional attributes of food products [43]. The addition of PSP resulted in an increase in the free sulfhydryl content of LF‐PSP complexes (Figure 6(d)), and the peak sulfhydryl levels for both LF and LF‐PSP observed at a pH of 3.0, which was 4.09 and 16.96 μmol/g, respectively, and both of them were significantly higher than those at other pHs. The main reason for the higher sulfhydryl content of LF‐PSP compared with that of LF could be that the interactions between LF and PSP facilitated the unfolding of LF, exposing more LF and PSP. Unfolding, exposing more free sulfhydryl groups. The alteration in sulfhydryl group content further demonstrates that the incorporation of PSP modifies the tertiary structure of LF [34]. The impact of this modification on the antioxidant or emulsifying capacity of LF and the LF‐PSP complex warrants further investigation.

### 3.10. Antioxidant Property Analysis

To investigate the antioxidant properties of LF and LF‐PSP complexes with different pH treatments, DPPH, ABTS, hydroxyl radical scavenging rate, and total reducing power of LF, PSP, and LF‐PSP complexes at different pH levels were detected.

DPPH is recognized as a stable free radical and is extensively utilized as a means to assess the free radical scavenging capability of antioxidants. The alcoholic solution of DPPH exhibits its peak absorption at 517 nm [44]. The method for scavenging DPPH involves reducing DPPH in the presence of antioxidants, forming the nonradical form DPPH‐H through the reaction [45]. Based on this principle, we determined the DPPH of LF, PSP, and LF‐PSP complexes at different pH as illustrated in Figure 7(a). The scavenging rate of DPPH for LF demonstrated a decreasing trend as the value of pH was increased (from 50.09% at pH 8.0%–30.45% at pH 8.0), aligning with our earlier observations [46]. In contrast, with the addition of PSP, the DPPH scavenging rate of LF was improved. The DPPH scavenging activity of the LF‐PSP complex was significantly higher than that of LF and PSP alone, reaching 81.79% at pH 4.0. This further demonstrates that pH changes affect the conformation of LF, and the presence of PSP stabilizes the structure of LF, enhancing its antioxidant activity.

FIGURE 7Antioxidant properties of LF, PSP, and LF‐PSP complexes: DPPH scavenging rate (a); ABTS scavenging rate (b); hydroxyl radical scavenging rate (c); reducing power (d).

**Figure:**
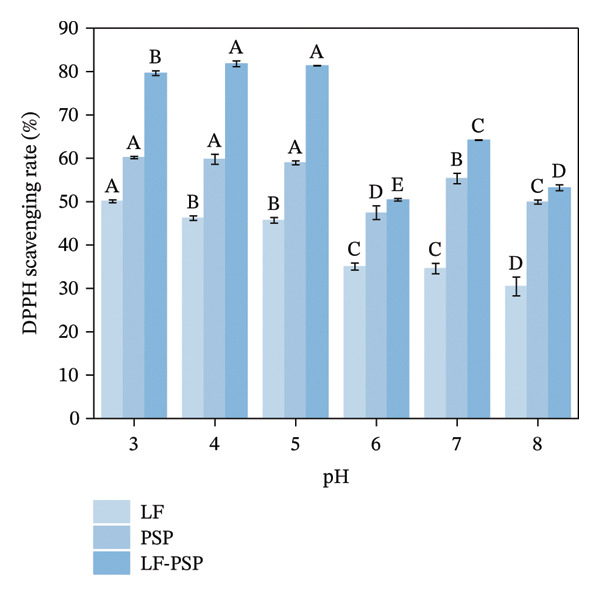
(a)

**Figure:**
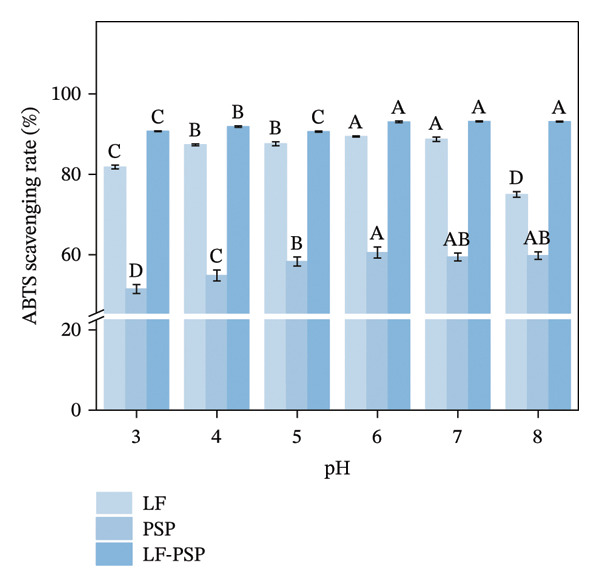
(b)

**Figure:**
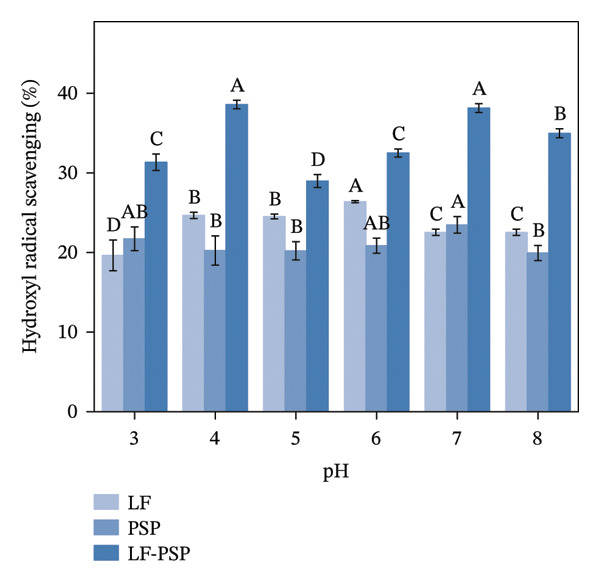
(c)

**Figure:**
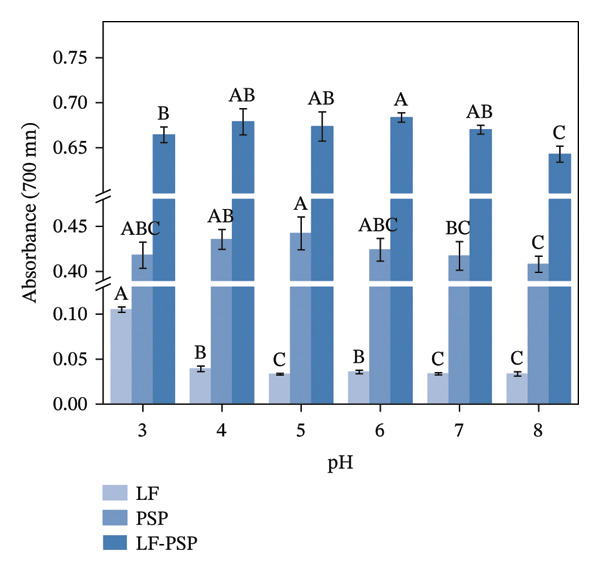
(d)

The ABTS technique is frequently employed to evaluate the overall antioxidant capacity of individual substances as well as intricate combinations derived from various plants [47]. Figure 7(b) illustrates the ABTS scavenging capabilities of the various samples. As with DPPH scavenging, the addition of PSP also enhanced the ABTS scavenging activity of the LF, resulting in significantly higher ABTS scavenging capacity than that of LF or PSP alone. The LF‐PSP complex achieved the highest ABTS scavenging rate of 93.18% at pH 7.0.

Hydroxyl radical is an extremely active free radical with high oxidizing properties. The mechanism by which hydroxyl radicals are produced generally requires the involvement of transition metal ions. When transition metal ions are absent, hydrogen peroxide tends to be fairly stable. However, in the presence of ions, such as ferrous iron or copper, hydrogen peroxide can produce highly reactive hydroxyl radicals via a reaction akin to the Fenton process. Therefore, molecules capable of inactivating metal ions through chelation may possess the ability to scavenge hydroxyl radicals [48]. Figure 7(c) illustrates that the scavenging rate of hydroxyl radicals by LF remained consistently under 30% across various pH levels, showing irregular variations with changes in pH. In contrast, the addition of PSP significantly increased the hydroxyl radical scavenging rate of LF, raising it above 30% in all cases, with a peak value of 38.58% at pH 4.0.

A direct relationship between the activity of antioxidants and their reducing power has been documented. The presence of reducing ketones is typically linked to these reducing properties, as they supply hydrogen atoms and demonstrate antioxidant effects by interrupting the chain reaction of free radicals [49, 50]. Additionally, reducing ketones can interact with certain peroxidation precursors, thereby inhibiting the formation of peroxides [51]. The reducing power of a compound can be an important indicator of its potential antioxidant activity. As shown in Figure 7(d), the LF‐reducing ability was poor across various pH levels. However, the introduction of PSP significantly enhanced the reducing power of LF, which reached its maximum at pH 6.0.

## 4. Conclusion

In this research, we successfully prepared a LF‐PSP complex via electrostatic interactions. Analysis using fluorescence spectroscopy reveals that LF forms a complex with PSP, and the introduction of PSP modifies the tertiary structure of LF. In addition, with the addition of PSP, the secondary structure of LF changed, mainly manifested as a decrease in the contents of β ‐ turn and random coil. These structural changes further influenced the functional properties of LF. We found that the addition of PSP could enhance the antioxidant activity of LF, but the effect was affected by the pH value. The DPPH radical scavenging ability, ABTS radical scavenging ability, hydroxyl radical scavenging rate, and reducing power reached their optimal levels under different pH conditions, indicating that there is no single pH value that can maximize all antioxidant indicators simultaneously. However, considering both physical stability and antioxidant activity, the LF‐PSP complex prepared within the pH range of 4–7 exhibited relatively good stability and high antioxidant capacity. Therefore, the complex prepared within this pH range has greater potential for practical applications. Overall, this research offers theoretical basis on the mechanisms involved in the interactions between PSP and proteins, as well as contributes to the progress of creating antioxidant‐enhanced foods. However, this study did not evaluate the antioxidant effect and mechanism of action of the complex in vivo, which limits the use of LF‐PSP complexes in food processing to a certain extent, and future studies will focus on their antioxidant mechanism and practical use in food production and processing.

## Author Contributions

Runru Xiang: investigation, formal analysis, methodology, and writing–original draft. Duoduo Zhang: methodology. Shunjie Kang: methodology. Jiwei Ding: methodology. Qin Shu: methodology. Guo Hao: methodology. Tao Ma: conceptualization, methodology, formal analysis, and writing–reviewing and editing. Yongfeng Liu: conceptualization, funding acquisition, supervision, and resources.

## Funding

This work was financially supported by the Shaanxi Science and Technology Plan Projects of China (2024NC‐GJHX‐30, 2024NC‐ZDCYL‐03‐07), Xi’an City Science and Technology Plan Projects of China (24NYGG0018), Xianyang City Science and Technology Plan Projects of China (L2024‐CXNL‐KJRCTD‐DWJS‐0010), Shaanxi Province Market Supervision Bureau Science and Technology Plan Projects of China (2024KY25), and the Fundamental Research Funds for the Central Universities in China (GK202506038, GK202306005).

## Conflicts of Interest

The authors declare no conflicts of interest.

## Data Availability

The data that support the findings of this study are available from the corresponding author upon reasonable request.
